# Irreversible Electroporation of Human Primary Uveal Melanoma in Enucleated Eyes

**DOI:** 10.1371/journal.pone.0071789

**Published:** 2013-09-05

**Authors:** Yossi Mandel, Shlomi Laufer, Michael Belkin, Boris Rubinsky, Jacob Pe'er, Shahar Frenkel

**Affiliations:** 1 Hansen Experimental Physics Laboratory, Stanford University, Stanford, California, United States of America; 2 Center for Bioengineering in the Service of Humanity and Society, School of Engineering and Computer Science, Hebrew University of Jerusalem, Jerusalem, Israel; 3 Ophthalmic Technologies Laboratory, Goldschleger Eye Institute, Tel-Aviv University, Sackler School of Medicine, Tel Hashomer, Ramat Gan, Israel; 4 Department of Mechanical Engineering, University of California Berkeley, Berkeley, California, United States of America; 5 Departments of Ophthalmology, Hadassah-Hebrew University Medical Center, Jerusalem, Israel; University of South Florida College of Medicine, United States of America

## Abstract

Uveal melanoma (UM) is the most common primary intraocular tumor in adults and is characterized by high rates of metastatic disease. Although brachytherapy is the most common globe-sparing treatment option for small- and medium-sized tumors, the treatment is associated with severe adverse reactions and does not lead to increased survival rates as compared to enucleation. The use of irreversible electroporation (IRE) for tumor ablation has potential advantages in the treatment of tumors in complex organs such as the eye. Following previous theoretical work, herein we evaluate the use of IRE for uveal tumor ablation in human *ex vivo* eye model. Enucleated eyes of patients with uveal melanoma were treated with short electric pulses (50–100 µs, 1000–2000 V/cm) using a customized electrode design. Tumor bioimpedance was measured before and after treatment and was followed by histopathological evaluation. We found that IRE caused tumor ablation characterized by cell membrane disruption while sparing the non-cellular sclera. Membrane disruption and loss of cellular capacitance were also associated with significant reduction in total tumor impedance and loss of impedance frequency dependence. The effect was more pronounced near the pulsing electrodes and was dependent on time from treatment to fixation. Future studies should further evaluate the potential of IRE as an alternative method of uveal melanoma treatment.

## Background

Uveal melanoma (UM) is the most common primary intraocular tumor in adults [1]. It is a highly malignant neoplasm, which threatens the patient with metastatic death, loss of the eye, and irreversible visual deficit. In the last two decades brachytherapy [2] and external irradiation (proton beam, gamma knife, etc.) are the most common treatment options for small to medium sized tumors with a success rate of about 90%, while enucleation remains the common treatment for large tumors. The collaborative ocular melanoma study (COMS) showed that patients who underwent either enucleation or brachytherapy had the same survival rates, and had the same risk for metastatic disease [3]. Brachytherapy, the most common globe sparing treatment modality for uveal melanoma, is delivered via radioactive plaques, mostly Ruthenium-106 (Europe) or Iodine-125 (USA). Complications of brachytherapy include neovascular glaucoma (with prevalence up to 45% in large tumors, 12% needed enucleation due to glaucoma), cataract (up to 68%, [4], [5], [6]) irradiation retinopathy with visual loss (up to 62%), retinal detachment and tears, optic nerve neuropathy (up to 46%, 5 years prevalence in large tumor [7], and others. The effect of this complication is a decrease of 2 lines of Snellen acuity in 26–62% of treated eyes [7]. Some patients undergo secondary enucleation [8] especially in large tumors. Lately, wall resection and endoresection have been added to the armamentarium of eye-preserving treatments for large tumors [9], [10]. Despite the great success in treating the primary tumor, patients have a risk of developing metastases over 20 years after the initial diagnosis [11]. The most common site for metastatic uveal melanoma is the liver [12]. The COMS identified 5- and 10-year cumulative metastasis rates of 25% and 34%, respectively, with 80% of the metastatic patients dying in the first year, and 92% in the first two years after the diagnosis of metastases [12].

Reversible electroporation is a technique used for membrane permeation by a high electric field, enabling high-level gene transfer [13], [14], [15], [16], [17] to specific organ tissue. The mechanism of the electroporation process is not fully understood; however, it is believed that the induced forces on membrane phospholipids and their motility can cause pore formation. The use of irreversible electroporation (IRE) for tumor ablation was only recently introduced by Rubinsky et al. in a series of theoretical and experimental studies [18], [19], [20], [21]. These studies showed that IRE induces tissue ablation, which is an independent non-thermal phenomenon. Since the electric field mainly disrupts the cell membrane, tissue ablation is limited to cells, and preserves the connective tissue scaffold as well as the blood vessel structures. These characteristics were found to be associated with a rapid regeneration process [21]. Another important characteristic of IRE is the clear-cut borders between affected and non-affected tissue, as was reported in the liver [21] and prostate. This contrasts with the gradual and indistinct margins found in thermal-based treatments [21]. The advantages of IRE as a minimally invasive treatment method make it an appealing choice for ocular tumor treatment because of the functional and histological complexity of the eye. Any surgical technique that is capable of protecting vulnerable structures (such as the lens, fovea, anterior chamber angle, and optic nerve) is important given the relatively limited intraocular surgical armamentarium.

Using a finite element simulation we had recently calculated [22] that above-threshold electrical field can be safely pulsed into a uveal melanoma tumor using a combination of external and internal or external only electrode configurations. The analysis found that low repetition pulsing rate is critical for prevention of eye temperature increase. Equipped with this knowledge we aimed at studying the feasibility of IRE for uveal melanoma by treating ex-vivo tumor with pulsed electrical field. Study aims were to evaluate the pathological changes caused by pulsed electrical field on the tumor and adjacent sclera and to characterize the effect of treatment on tumor electrical conductivity.

## Results

### Histopathological Findings

**Figure 1:**
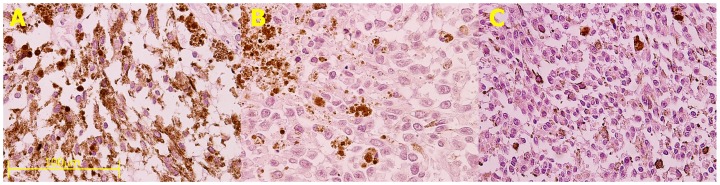
The effect of IRE on uveal melanoma tumor in eye enucleated from an 81-year-old female presented with an extra-large uveal melanoma (15.8×18.3×13.4 mm). The eye was enucleated and opened fresh in the operating room. A piece of the tumor from the callot was divided for different IRE treatments. **A**. Tumor was treated by 200 pulses of 1000 V/cm, 50 µs followed by incubation for 3 hours in 37°C before fixation in 4% formalin. This tumor shows large areas of empty spaces, probably caused by membrane disruption and loss of cellular cytoplasm. Nuclei appear to be less affected by the electric field and are preserved within empty cytoplasm. **B**. A piece of the tumor was incubated for 3 hours in 37°C without any other treatment. Histopathologic evaluation shows no evidence of damage to the membranes. **C**. This piece of the tumor was treated as the piece in Figure 1A, but fixed immediately in 4% formalin. This specimen shows a mixed cell type (spindle and epithelial cells) packed densely together with no apparent effect of the treatment. (Hematoxylin-eosin staining, magnification ×40, bar equals 100 µm for all the panels).

IRE treatment caused characteristic loss of cellular cytoplasm probably due to membrane breakdown (**Fig. 1A**). In most cells, the nuclei remained unaffected and surrounded by the cells' membrane in an empty cytoplasm. The ablation effect of the treatment was extensive and most tumor cells were affected. The effect required time to become apparent and was not noticed when the tumor was immediately fixed after treatment (**Fig. 1C**). A control, untreated specimen (**Fig. 1B**) showed no changes following incubation in medium for 4 hours at 37°C (as the treated tumor in Fig. 1A) and depicted a pathological appearance similar to that of an untreated tumor fixed immediately following enucleation.

**Figure 2:**
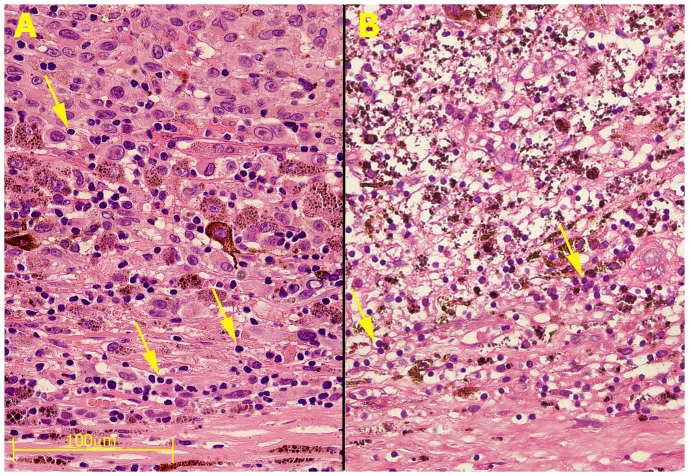
The effect of IRE on uveal melanoma tumor in eye enucleated from a 67-year-old woman presented with a local recurrence of uveal melanoma 27 months after initially successful treatment with brachytherapy (Ru-106). The eye was enucleated and opened in the operating room to remove a piece of the tumor for IRE. That piece was treated by 100 pulses of 1000 V/cm, 50 µs, and incubated for 3 hours in medium in 37°C before fixation. **A**. A pupil-optic nerve section that was not treated and was fixed immediately after the eye was opened. The slide shows (from the bottom up): the sclera, an area with some inflammatory cells and mostly dead tumor cells from prior brachytherapy, and viable epitheloid cells on top. **B**. IRE treated tumor in a matching section to panel A. Note that except for a few apparently unaffected lymphocytes (arrows), the entire area contains no living cells and the area of viable epitheloid cells was completely ablated. (Hematoxylin-eosin staining, magnification ×40, bar equals 100 mm for both the panels).

Similar ablation of tumor cells was achieved in another case following treatment with only 100 pulses of 1000 V/cm (**Fig. 2**). This case was treated previously by brachytherapy. However, 2 years after the treatment there was a local recurrence and the eye was enucleated. **Fig. 2A** shows a specimen that was not treated with IRE and depicts some viable living tumor cells within the brachytherapy scar (adjacent to the sclera) and a mass of viable epitheloid cells in the area further away from the sclera toward the center of the eye. In contrast, (**Fig. 2B**), IRE caused ablation of cells situated further away from the sclera. Of interest is the complete destruction of the epitheloid tumor cells upon IRE treatment in the treated tumor (**Fig. 2B**) as compared to the untreated specimen (**Fig. 2A**). Of importance, apparently, the sclera was not affected by treatment since it is mainly composed of connective tissue which is not affected by the non-thermal ablation, as opposed to cellular membrane [21].

**Figure 3:**
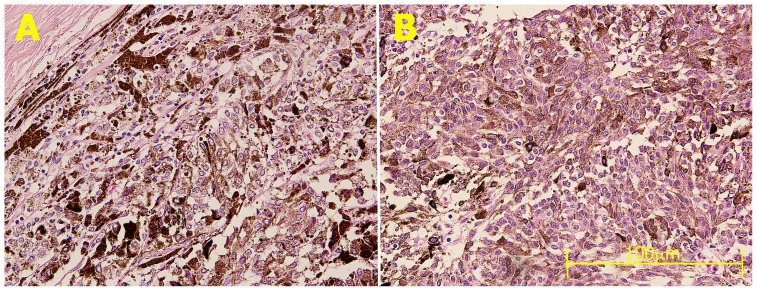
The effect of IRE on uveal melanoma tumor in primary eye enucleated eye from a 45-year-old woman presented with an extra-large choroidal melanoma (16.4×16.8×17.0 mm). The eye was enucleated and opened fresh in the operating room. A piece of the tumor from the callot was divided for different IRE treatments (200 pulses of 2000 V/cm at 50 µs). **A** An area adjacent to the sclera and the electrodes where the tumor cells' nuclei appear large and foamy and there is loss of the cytoplasm caused by membrane disruption. **B**. An area far away from the electrodes that were applied to the sclera was seemingly unaffected by treatment, probably because the electric field was lower than ablation thresholds. Note that lymphocytes are not affected by treatment (arrows) at area where tumor cells were ablated (Hematoxylin-eosin staining, magnification ×40, bar equals 100 µm for both panels).

In another case of a primary enucleation of an eye with an extra-large tumor in a 45-year-old woman that was treated with 200 pulses of 2000 V/cm at 50 µs, tumor cells ablated by pulse treatment showed foamy nuclei and loss of cytoplasm. In this tumor sample only the area close to the sclera was affected (Fig. 3A) while areas far away from the sclera were less affected (Fig. 3B), demonstrating the importance of electrode-tumor proximity for treatment success.

### Effect of Treatment on Tumor Bioimpedance

**Figure 4:**
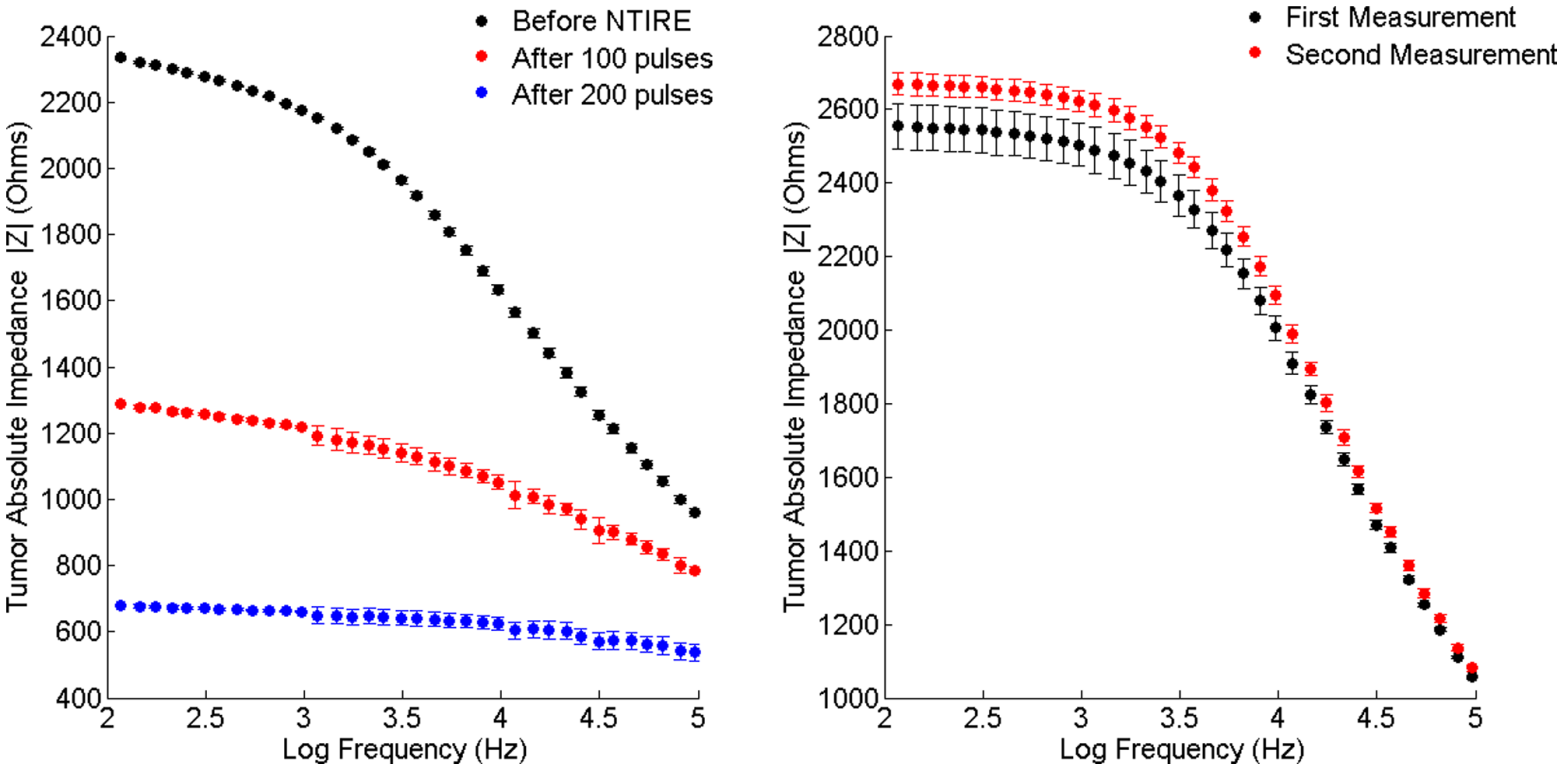
Effect of pulse electrical field on tumor absolute impedance. **A** Absolute impedance of uveal melanoma tumor from an eye enucleated from a 74-year-old female. Impedance was measured at frequencies of 100 Hz–100 kHz. Pulsed electrical field caused a 4-fold decrease in absolute tumor impedance and loss of frequency relaxation, suggestive of membrane breakdown. **B** Impedance stayed stable in a control tumor specimen kept in similar condition with no treatment.

Pulse treatment caused a characteristic change in bioimpedance measurement of the tumor. Following IRE treatment tumor impedance was reduced in all four tumor samples measured in this study. The average decrease in low frequency impedance was 3.4-fold, significantly larger as compared to the 1.9-fold impedance decrease at high frequency (student paired t-test 0.016). Fig. 4 shows a characteristic impedance change following IRE treatment. Absolute impedance of the tumor before treatment (Fig. 4A, black trace) significantly decreased with frequency (the tumor's absolute impedance at 100 Hz and 100 kHz were 2334 Ohm and 960 Ohm, respectively). This phenomenon, sometimes referred to as beta relaxation, is caused by the capacitive effect of the lipid cell bilayer membrane and is characteristic of bioimpedance of cellular tissues. Following 100 pulses of 2000 V/cm (Fig. 4a, red trace), the impedance dropped by a factor of about 2 and following another set of 100 pulses (Fig. 4a, blue trace) there was again a two-fold decrease in impedance. Further, the electrical pulses caused loss of the impedance frequency dependency, suggesting loss of capacitance component which was caused by cell membrane breakdown. Interestingly, tumor impedance dropped immediately following treatment, while there was only minimal histological evidence for treatment in this case, probably because it was fixed early after treatment. The impedance stayed stable in a control, untreated tumor specimen (Fig. 4b) which was maintained untreated under similar culture conditions.

## Discussion

In this paper we show for the first time that short electric pulses were effective in ablation of uveal melanoma tumors *ex-vivo*. Ablation caused distinct histopathological features suggestive of cell membrane permeation and rupture. The histological findings are similar to results described following IRE in other solid organs [19], [23], [24] where extensive loss of cytoplasm occurred several hours after pulsing the cells.

The main mechanism for cellular ablation in IRE is an irreversible membrane disruption, causing cell cytoplasm to exit the cells through large pores. This process occurs immediately following electric pulse, as reported by Gehl et al [25] or Bobanovic [26] in studying the dynamics of small molecules uptake after electric pulsing. Interestingly, the histopathological changes in our experiment were dependent on the duration between treatment and fixation and were not apparent when tumors were immedately fixed following treatment. This could be explained by the enhancement of membrane breakdown via entrance of extracellular fluid into the cell following partial loss of membrane integrity, or by other time-dependent processes. The optimal time of fixation post IRE is not clear, since on the one hand, the effect of IRE is expected to appear after a few hours post treatment, while on the other hand, prolonged incubation in culture media can induce histopthological changes in the incubated tumor. Nevertheless, the small number of cases available for this study did not enable comparing various durations of time between treatment and fixation.

Another mechanism to explain the observed cellular death is induction of apoptosis. Apoptosis was reported to be the mechanism of cell death following high electrical field pulsing of a lymphoblast culture [27]. An *in vivo* experiment in mouse sarcoma tumor model found evidence for apoptosis (TUNEL staining) as early as 5 minutes after IRE treatment [23]. Similarly, Tracy reported apoptosis at 1 hour following *in vivo* IRE in swine renal tissue [28]. Nanosecond pulses are also known to induce apoptosis by affecting the intracellular membrane without affecting the cell membrane [29], [30], further suggesting that the ablative effect of IRE is a complex process. Nevertheless, this study was not designed to explore the role of apoptosis in uveal melanoma IRE ablation and further studies should address this issue.

The membrane potential of a cell exposed to a homogenous electric field is linearly correlated with cell diameter [31]. The average uveal melanoma cell radius is 15–23 microns [32] whereas a typical lymphocyte radius is 7 µm [33]. Thus, when exposed to the same electrical field, the membrane potential of uveal melanoma cells will be 2–3 times higher than that of the lymphocyte [31]. Consequently, the threshold for membrane breakdown in lymphocytes is 2–3 times higher than that of uveal melanoma cells. It therefore comes as no surprise that in our samples, lymphocytes were not unaffected by the electric pulses that damaged the melanoma cells (Figs. 2A,B).

It is clinically important that the sclera was not affected by the electric pulse treatment. Similar results were observed by our group in sclera of rats exposed to high electric field pulsing [34]. The relative resistibility of sclera to electric pulsed treatment is caused by the selectivity of IRE to cell membranes while sparing connective tissues. This selectivity makes IRE favorable in areas where sparing of blood vessels, nerve or connective tissue is critical, such as in pancreatic tumor [35], bile duct and prostate [36].

The frequency dependence of pre-treatment tumor impedance (**Fig. 4A**, upper trace) is characteristic for dense cellular tissues and is caused by the capacitance properties of the bilayer cell membrane. Tissue electrical conductivity is often modeled as a parallel combination of a capacitor, representing cell membrane and a resistor, representing the extra-cellular fluids. At lower frequencies current will flow mainly through the extracellular space as the capacitor effect of the membrane will produce high resistivity. In contrast, at higher frequencies, current will pass through the membrane because of its capacitive coupling, and the total impedance of the tissue is reduced. This frequency dependence of the impedance is called beta dispersion or relaxation. The electric pulse treatment caused a dose response drop in tumor impedance and loss of frequency relaxation; both are caused by cell membrane permeation. The results are in good agreement with prior studies [24], [37], [38] where a drop of up to 4-fold was observed following IRE treatment. We found that immediately following treatment and early fixation, there was a significant decrease in impedance while only minimal histopathological changes were found. This could be explained by the immediate effect of electrical pulses on membrane integrity as opposed to the complex pathological process leading to complete membrane disruption, apoptosis and cell loss, occurring hours following treatment.

We had recently reported that low tumor conductivity is associated with higher intratumor electrical field and increased treatment efficiency [22]. The increase in tumor conductivity following treatment is expected to decrease treatment efficiency. Further, the increased tumor conductivity is also associated with increased heat production. Taken together, the effect of treatment on tumor conductivity should be taken into consideration when designing a treatment plan when uveal melanoma or other tumors are treated by IRE. On the other hand, the change in conductivity can be used for treatment monitoring as we previously suggested [39], [40].

Given the small availability of cases for the study, the current study was not designed to determine the threshold electric field for uveal melanoma IRE. Nevertheless, IRE was achieved by 100 pulses of as low as 1000 V/cm at 50 µs pulse duration. The results are in agreement with prior reports [23] where IRE treatment was applied in a subcutaneous sarcoma tumor model in mice. Similar results were found [20], [39] when application of an electric field with an intensity of 1500 V/cm and a pulse duration of 300 microseconds induced cell ablation in primary human hepatocellular carcinoma cells. More recently, however, Rubinksy et al [36] reported that 90 pulses of 250 V/cm induced total ablation of prostate cancer cells *in vitro*. Thus, the optimal pulse parameters for tumor ablation in various tissues are still not known.

In conclusion, in this study we demonstrated that uveal melanoma tumors can be ablated *ex vivo* by short electrical pulses with clinical applicable electrical field. Tumor conductivity increased significantly during treatment, calling for proper treatment planning and monitoring. Further studies should be done in order to estimate the potential role of IRE as a new globe-sparing treatment modality for uveal melanoma.

## Methods

### Patient selection, history and clinical evaluation

**Figure 5:**
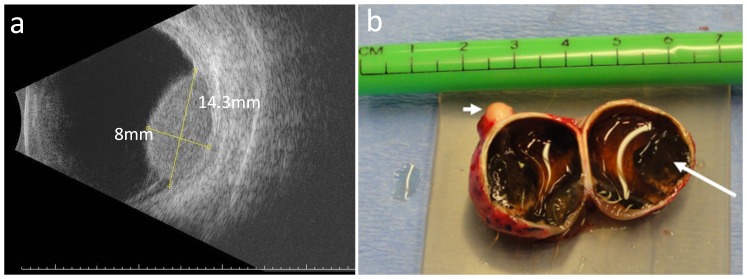
Clinical evaluation and surgical procedure for uveal melanoma tumors. a. B-Scan ultrasonography imaging of an eye of a 68-year-old patient shows a dome-shaped medium reflective posterior (choroidal) tumor with a largest basal diameter of 14.3 mm and a height of 8 mm, with shallow rimming retinal detachment. b. Following gross examination an enucleated eye was cut in two and one half was clinically processed while the other was used for the experiment. The long arrow points to tumor, the short arrow points to optic nerve.

Five eyes of female patients with a mean age (±SD) of 70.8±15.1 years (range 46 to 83 years) undergoing enucleation due to uveal malignant melanoma were selected for participation in the study. Average largest basal diameter was 16.09±1.74 mm (range 13.6–18.3 mm) and the average tumor height was 10.3±4.0 mm (range 6.0–14.7 mm). Two cases extended from the ciliary body to the choroid, one was in the anterior choroid and the remaining two where located in the posterior pole. Two cases were enucleated following local recurrence after brachytherapy, while in the remaining three cases enucleation was the first treatment with no prior surgical or other treatment. Uveal melanoma was diagnosed based on a clinical examination by an expert ocular oncologist (JP or SF) coupled with the ultrasonic (US) appearance of those tumors. **Fig. 5a** shows a characteristic US imaging of a 68-year-old patient at the time of diagnosis with uveal melanoma. The experiment was approved by the Hadassah Medical Center IRB and patients signed an informed consent for using part of the enucleated material for the experiment.

### Surgical Procedure

Enucleated eyes were grossly evaluated in the operating room and transillumination was used to identify the tumor margins. A pupil-optic nerve section was cut through the shadow of the tumor (**Fig. 5b**). Half of the eyeball was immediately fixed in formaldehyde for clinical histopathological evaluation while the other half was used for the experiment. The sclera around the tumor base was resected by surgical scissors leaving a rim to mechanically support the tumor. In some cases, the tumor was further divided into several specimens, which were subjected to different treatment parameters or were left as controls. Tumor specimens were transferred within 1 hour post enucleation to culture media and kept in 37°C until bioimpedance measurements and IRE were performed. The culture media was composed of Minimum Essential Medium-Eagle (MEM-E), Earle's salts base, without L-glutamine with 10% Fetal Bovine Serum (FBS), L-Glutamine, Penicillin-streptomycin, and non-essential amino acids (Biological Industries, Beit Ha'emek, Israel). This medium is used for growing uveal melanoma cell lines in culture [41].

### Bioimpedance Measurements and Pulse Treatment

**Figure 6:**
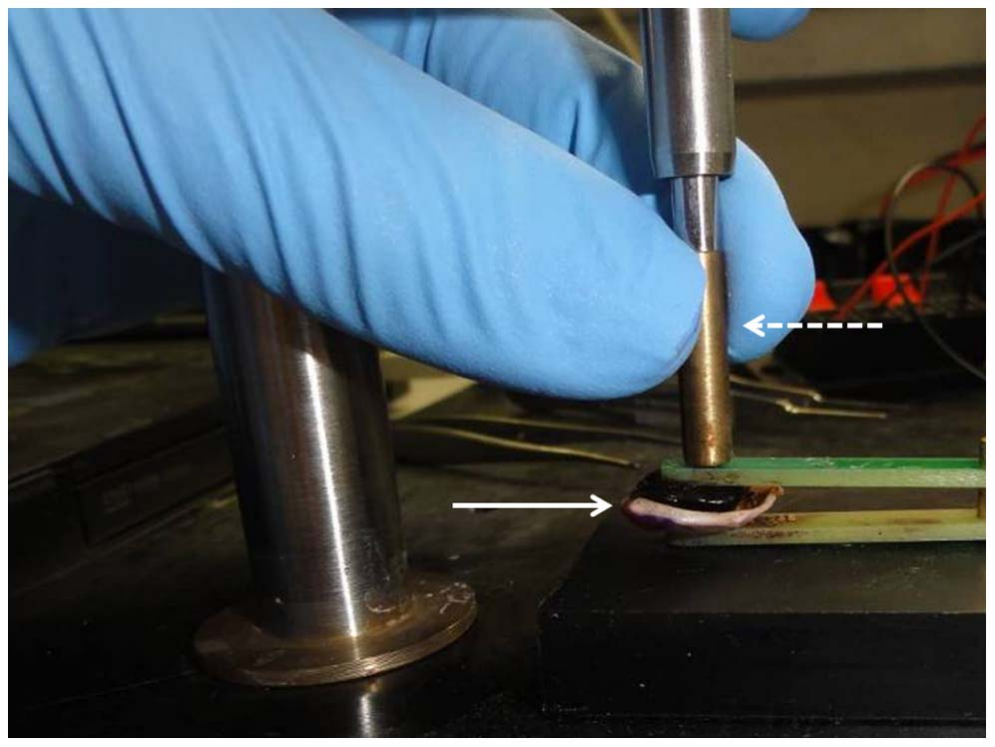
Experimental setup for *ex vivo* uveal melanoma impedance measurement and pulse treatment. Excised uveal-tumor specimen (solid line arrow) was placed between customized electrodes used for both impedance measurement and pulse treatment. Specimen height was measured by a micrometer (dashed line arrow) for calculation of electric field.

Samples were positioned in a customized measurement device in which electrical properties were measured under constant pressure and the distance between the electrodes was measured by a micrometer (**Fig. 6**). Impedance measurements and electrode configurations were reported previously by our group [24], [42]. In short, the impedance data were collected using an electrochemical analyzer chi604c (CH Instruments, Inc, Austin, TX, USA) at 11 equal logarithmically spaced frequencies between 100 Hz–100 KHz. In order to reduce tissue-electrode interface error, we used four electrodes configuration geometry with external annular electrode of 4 mm diameter and a central 0.5 mm disc. Electrodes were made of gold coated with platinum black to further reduce the electrode-tissue interface and were built on two parallel plates produced by using Printed Circuit Board (PCB) technology. Four out of five tumors showed good quality bioimpedance data and are analyzed in this paper.

Treatment was given through the same electrode system used for impedance measurements. Fifty to 200 pulses of 50 microseconds with electric field of 1 kV/cm–2 kV/cm were applied at a repetition rate of 0.5 Hz using an electroporator (BTX ECM 830, Harvard apparatus, Holliston, MA, USA). Current was measured by a scope (LeCroy Waverunner 64xi) with a LeCroy AP105 Current Probe. Following pulse treatment bio-impedance measurements were taken again and the tumor specimens were left in the medium for 1–6 hours at 37°C in an incubator, after which they were fixed in 4% formaldehyde for routine tissue processing. In two cases the time from treatment to fixation was 1.5–2 hours. In the other specimens, the time in medium was prolonged to 5 hours in order to enable post treatment processes to take place (see *Discussion*). In all cases, controlled untreated specimens were always kept in the same conditions as the treated specimens until fixation.

### Fixation Process

The specimens were fixed in 4% formalin, and then routinely processed. Tissue sections of 4 µm thickness were prepared and stained with hematoxylin and eosin.
